# Duties, resources, and burnout of antibiotic stewards during the coronavirus disease 2019 (COVID-19) pandemic

**DOI:** 10.1017/ash.2021.200

**Published:** 2021-11-05

**Authors:** Valerie M. Vaughn, Guinn E. Dunn, Jennifer K. Horowitz, Elizabeth S. McLaughlin, Tejal N. Gandhi

**Affiliations:** 1 Division of General Internal Medicine, Department of Internal Medicine, University of Utah School of Medicine, Salt Lake City, Utah, USA; 2 Division of Health System Innovation & Research, Department of Population Health Science, University of Utah School of Medicine, Salt Lake City, Utah, USA; 3 Division of Hospital Medicine, Department of Internal Medicine, Michigan Medicine, Ann Arbor, Michigan, USA; 4 Department of Internal Medicine, University of Utah School of Medicine, Salt Lake City, Utah, USA; 5 Division of Infectious Diseases, Department of Internal Medicine, Michigan Medicine, Ann Arbor, Michigan, USA

During the COVID-19 pandemic, antibiotic stewardship leaders (ie, stewards) were frequently called upon to help coordinate hospital pandemic responses.^1^ We surveyed 51 antibiotic stewardship leaders to identify how the roles of stewards changed during the pandemic and how these changes affected workload, antibiotic stewardship activities, and steward burnout.

## Methods

### Data collection

Surveys were e-mailed to all 51 Michigan Hospital Medicine Safety Consortium (HMS) hospitals on December 8, 2020. Hospitals were asked to have their “antibiotic stewardship lead” (defined by each hospital) complete the survey. In 2019, 77% of HMS hospitals identified an infectious diseases physician as their stewardship leader.

### Survey measures

We followed the American Association for Public Opinion Research’s “Best Practices for Survey Research.”^2^ Most survey questions were designed specifically for this survey; burnout questions were adapted from the literature.^3^ We obtained input on survey design from stewards and survey experts.

The survey assessed 5 domains related to COVID-19 and antibiotic stewardship: (1) additional COVID-19–related duties for stewards, (2) changes in full-time equivalents (FTE, the workload of a full-time employee) for stewardship, (3) the impact of COVID-19 on traditional stewardship activities, (4) new COVID-19 or telestewardship initiatives, and (5) stewardship clinician burnout. (A full survey is listed in the Appendix.) Responses were a mixture of free text, multiple choice, and Likert scale options. Hospital characteristics were obtained from national databases. (Data sources are listed in the Appendix.)

### Data analysis

Survey responses were characterized as either percentage of respondents or median and inter-quartile range (IQR). Free-text responses to the question on increased hours per week were often given as ranges (eg, 10–20 additional hours per week); we report these responses as ranges with average low and high responses. For Likert-scale responses to the burnout questions, we report raw responses as well as the percentage of respondents who scored ≥4 on combined emotional exhaustion and depersonalization questions, indicating a positive screen for burnout.^3^

## Results

The survey response rate was 100% (51 of 51), with lower response rates for burnout questions (75%–80%). Surveyed hospitals had a median of 310 beds (IQR, 189–422); 45 (88%) of 51 were largely academic and 44 (86%) were nonprofit. A median of 1.2 FTEs (IQR, 0.7–3.5) were dedicated for antibiotic stewardship prior to the COVID-19 pandemic (Appendix).

Antibiotic stewardship leaders reported a median of 5 new duties (IQR, 3–8) for the stewardship team that required a major effort (Fig. 1A). Despite the growing list of duties for stewards since the COVID-19 pandemic, only 4 (8%) of 51 respondents reported increased FTEs, whereas 9 (18%) reported decreased FTEs. As a result, 42 (82%) of 51 respondents reported that they now worked, on average, 9–11 more hours per week than before the pandemic; only 9 (18%) reported no change in hours.

**Fig. 1A.**
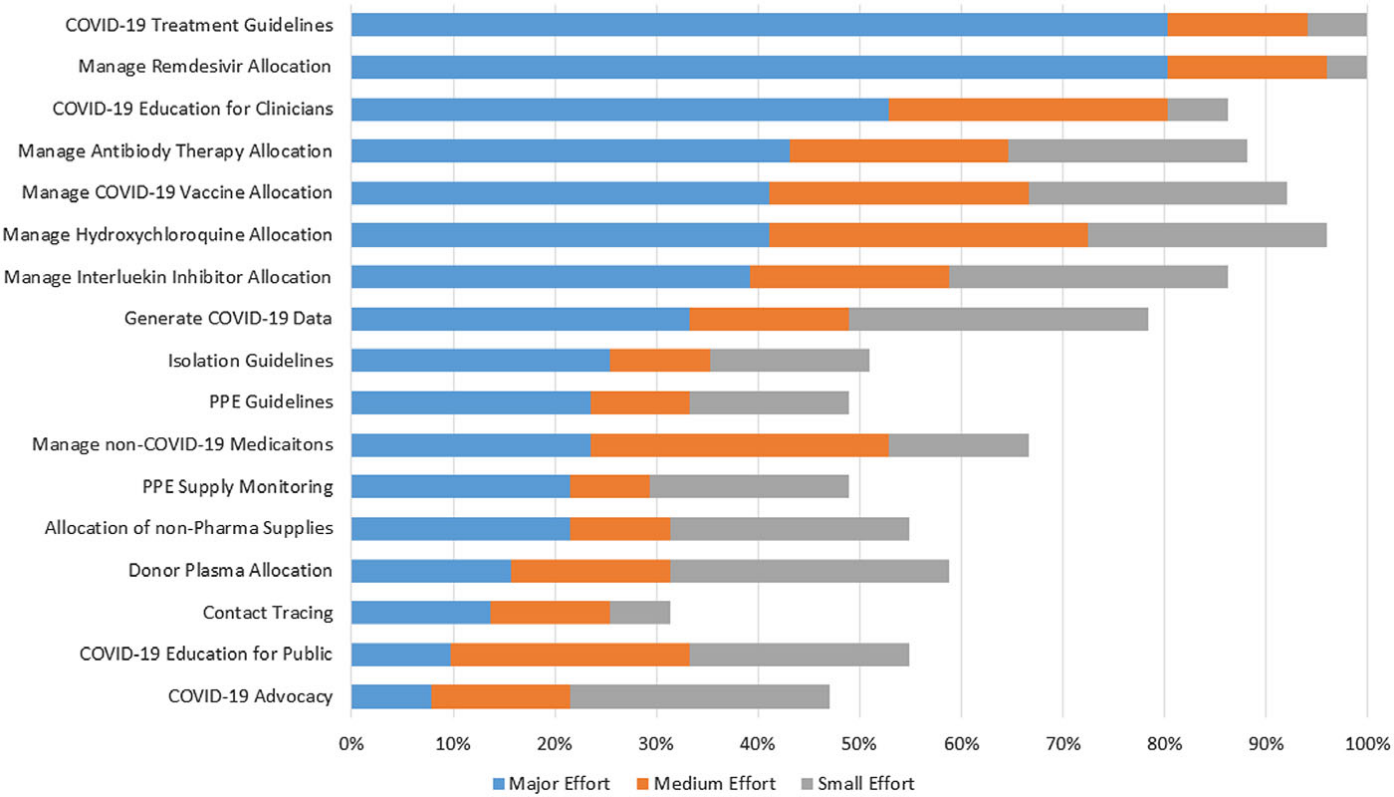
Additional Duties Reported by Stewards (N = 51 antibiotic steward leaders). “Since the COVID-19 pandemic, has the antibiotic stewardship team been called to perform any of the following duties. If yes, please mark as small, medium, or major effort.”

When asked how traditional antibiotic stewardship duties were affected by the COVID-19 pandemic, 37 (73%) of the 51 respondents reported that it had somewhat decreased their team’s ability to perform stewardship and 13 (25%) respondent that it had strongly decreased their team’s ability to perform stewardship. Only 1 respondent reported no effect. Overall, Stewardship leaders reported that a median of 5 items (IQR, 3–7) affected antibiotic stewardship. Among them, 40 (78%) cited stewardship time now spent on COVID-19 (Fig. 1B).

**Fig. 1B.**
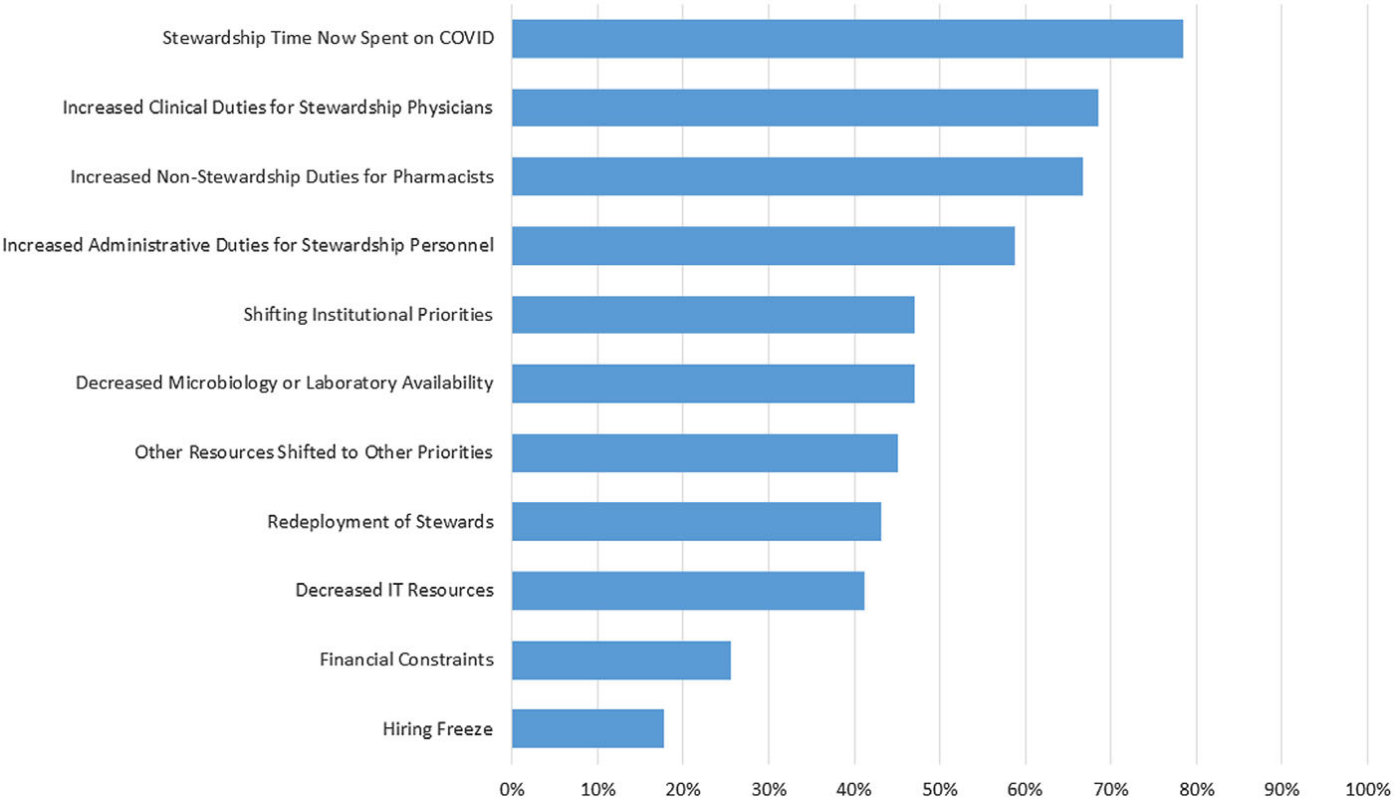
Items Due to COVID-19 Impacting Stewardship Initiatives (n = 51 antibiotic stewardship leaders). Responses shown to the question, “Since the COVID-19 pandemic have any of the following affected your ability to perform antibiotic stewardship activities.”

As nonstewardship COVID-19–related duties increased, so did COVID-19–necessitated stewardship: 42 (82%) respondents reported monitoring antibiotic use in hospitalized patients with COVID-19 and 25 (49%) reported new COVID-19–related stewardship interventions. Only 1 respondent (2%) reported initiating each of the following since the pandemic began: monitoring antibiotic use during televisits, telestewardship activities, and tele-infectious diseases activities. Two stewards (4%) reported starting a COVID-19 hotline.

Of 40 respondents, 18 (45%) reported feeling emotionally drained from their work a few times per week and 6 (15%) reported feeling emotionally drained from their work daily. Of 38 who responded when asked how often they felt as though they treated some patients as if they were impersonal objects, 1 (2.6%) indicated a few times per week and 4 (10.5%) indicated daily (eFig. 1). When combined as a 2-question score, 27 (71%) of 38 had a score ≥4, corresponding to potential burnout. Nearly all respondents indicated that burnout symptoms were higher than before the pandemic; among 41 respondents, 14 (34%) indicated that burnout symptoms were much higher; 25 (61%) of 41 indicated that burnout symptoms were somewhat higher; and only 2 (4.9%) indicated no change.

## Discussion

In a survey of antibiotic stewardship leaders at 51 Michigan hospitals, stewards reported substantially more duties related to COVID-19, similar or fewer resources, more work hours, decreased ability to carry out antibiotic stewardship initiatives, and more symptoms of burnout than before the pandemic.

COVID-19 substantially affected the roles and activities of antibiotic stewardship leaders. Antibiotic stewardship is critical for addressing antibiotic resistance—one of the top threats to health worldwide.^4,5^ In the present study, antibiotic stewards reported being overworked with limited resources and noted their ability to conduct both traditional and COVID-19–related antibiotic stewardship has been limited. Major barriers include diversion of personnel and other resources to managing the COVID-19 pandemic.

Although many have called upon antibiotic stewardship teams to help with the pandemic, these requests for help have been far more sustained than originally anticipated, and they do not appear to have been accompanied by increased resources. Instead, stewards reported working more hours. The high percentage of burnout symptoms is alarming and matches what is seen with many other frontline providers.^6,7^

The limitations of the study include self-reported data and lack of survey validation. The survey was administered in a single state when hospitalizations for COVID-19 were particularly high. Thus, we may have overestimated duties and burnout. The strengths of this study include hospital diversity and high response rate.

In conclusion, like many of those called upon to respond to COVID-19, antibiotic stewardship leaders are struggling and need additional support.

## Supplementary material

For supplementary material accompanying this paper visit https://doi.org/10.1017/ash.2021.200. click here to view supplementary material
