# Supplementary material for: Duties, resources, and burnout of antibiotic stewards during the coronavirus disease 2019 (COVID-19) pandemic
Source: Antimicrob Steward Healthc Epidemiol. 2021 Nov 5;1(1):e39. doi: 10.1017/ash.2021.200 (PMC9495633; doi:10.1017/ash.2021.200)
Supplement: Supplementary file 1 [file S2732494X2100200Xsup001.docx]

**Duties, Resources, and Burnout of Antibiotic Stewards During the COVID-19 Pandemic**

**Valerie M. Vaughn MD, MSc, Guinn E. Dunn MD, Jennifer K. Horowitz MA, Elizabeth S. McLaughlin MS, RN, Tejal N. Gandhi MD**

**Effect of COVID-19 on Antibiotic Stewardship Questionnaire Pages 2-4**

**Data Sources for Hospital Characteristics Page 5**

**eTable 1: Hospital Characteristics Page 6**

**Effect of COVID-19 on Antibiotic Stewardship Questionnaire**

1. Prior to the COVID19 pandemic, how many FTE's for total staff did you have available for antibiotic stewardship activities. (free text)
2. Since the COVID19 pandemic, have FTEs available for antibiotic stewardship activities:

- Increased
- Decreased
- Stayed the same

*If Increased*: You stated that since the COVID19 pandemic FTEs available for antibiotic stewardship increased. Please indicate how many were increased and the reasons for adding additional FTEs. (free text)

*If Decreased*: You stated that since the COVID19 pandemic FTEs available for antibiotic stewardship decreased. Please indicate how many FTEs were decreased and the reasons for decreasing. (free text)

1. Since the COVID-19 pandemic, have any of the following affected your ability to perform antibiotic stewardship activities? (check all that apply)

- Hiring Freeze
- Increased clinical duties for antibiotic stewardship physicians
- Increased non-stewardship duties for antibiotic stewardship pharmacists
- Redeployment of antibiotic stewards to other duties (e.g., stewardship pharmacists or physicians to non-stewardship roles)
- Decreased microbiology/laboratory resources/availability
- Decreased IT resources/availability
- Other resources to other priorities
- Shifting institutional priorities
- Financial constraints
- Increased administrative duties for antibiotic stewardship personnel to do non-COVID stewardship work
- None of the above

1. Overall, how has your team’s ability to perform antibiotic stewardship been impacted by the pandemic?

- Strongly decreased our ability to perform antibiotic stewardship
- Somewhat decreased our ability to perform antibiotic stewardship
- No effect on our ability to perform antibiotic stewardship
- Somewhat increased ability to perform antibiotic stewardship
- Greatly increased ability to perform antibiotic stewardship

1. Since the COVID-19 pandemic, has the antibiotic stewardship team been called on to perform any of the following duties? If yes, please mark as small, medium, or major effort.

|  | No Effort | Small Effort | Medium Effort | Major Effort |
| --- | --- | --- | --- | --- |
| Create COVID-19 treatment guidelines | ○ | ○ | ○ | ○ |
| Manage drug allocation- Remdesivir | ○ | ○ | ○ | ○ |
| Manage drug allocation- Hydroxychloroquine | ○ | ○ | ○ | ○ |
| Manage drug allocation- Interleukin inhibitors (e.g., Tocilizumab) | ○ | ○ | ○ | ○ |
| Manage drug allocation- Donor plasma | ○ | ○ | ○ | ○ |
| Manage drug allocation- Antibody therapy | ○ | ○ | ○ | ○ |
| Manage drug allocation- COVID vaccine | ○ | ○ | ○ | ○ |
| Manage drug allocation- Other non-COVID medications | ○ | ○ | ○ | ○ |
| Manage allocation- on-pharmaceutical supplies (e.g., PPE) | ○ | ○ | ○ | ○ |
| Additional infection prevention duties- PPE supply monitoring | ○ | ○ | ○ | ○ |
| Additional infection prevention duties- PPE guidelines | ○ | ○ | ○ | ○ |
| Additional infection prevention duties- Isolation guidelines | ○ | ○ | ○ | ○ |
| Additional infection prevention duties- Contact tracing | ○ | ○ | ○ | ○ |
| COVID education (to clinicians) | ○ | ○ | ○ | ○ |
| COVID education (to public) | ○ | ○ | ○ | ○ |
| COVID data generation (e.g., modelling, outcome data) | ○ | ○ | ○ | ○ |
| COVID advocacy (e.g., government, payor) | ○ | ○ | ○ | ○ |
| Other | ○ | ○ | ○ | ○ |

1. On average, how many additional hours per week is each member of your stewardship team now spending on work-related duties than before the COVID-19 pandemic? (e.g., if you worked 40 hours per week before, and now work 50, please write 10 hours). If not additional hours, please indicate “No additional time”.

|  |
| --- |

1. Since the COVID-19 pandemic, have you begun any of the following:

- Monitoring antibiotic use during tele-visits
- Monitoring antibiotic use in hospitalized patients with COVID-19
- Implemented stewardship interventions to improve antibiotic use in hospitalized patients with COVID-19
- Tele-stewardship activities
- Stewardship hotline
- COVID hotlines (run through antibiotic stewardship)
- Tele-ID (run through antibiotic stewardship
- None of the above.

1. We are trying to obtain a sense of the level of burnout present in stewardship clinicians. If you are uncomfortable answering, please skip the following two questions. Please answer the following two questions **for yourself**.

|  | Every day | A few times a week | Once a week | A few times a month | Once a month | A few times a year | Never |
| --- | --- | --- | --- | --- | --- | --- | --- |
| a) I feel emotionally drained from my work | ○ | ○ | ○ | ○ | ○ | ○ | ○ |
| b) I feel emotionally drained from my work | ○ | ○ | ○ | ○ | ○ | ○ | ○ |

1. How does the frequency of these emotions differ than before the pandemic?

- Much higher
- Somewhat higher
- About the same
- Somewhat lower
- Much lower

**Data Sources for Hospital Characteristics**

Academic hospital status

- Obtained from the American Medical Association’s FREIDA (Fellowship and Residency Electronic Interactive Database) Institution Directory
- Retrieved 2/2/2021 from https://freida.ama-assn.org/institution?page=9&filter=M

Hospital profit status

- Obtained from data.medicare.gov
- Retrieved from 06/03/2020 from <https://data.medicare.gov/widgets/xubh-q36u>

Hospital bed size

- Obtained from the 2015 Michigan Certificate of Need Annual Survey, Basic Total Licensed Beds Utilization Statistics
- Retrieved 3/21/2017 from <http://www.michigan.gov/documents/mdhhs/Report_011_-_Licensed_Beds_in_Hospitals_by_County_538170_7.pdf>

**eTable 1**. Hospital Characteristics, N=51 Hospitals

| **Variable** |  |
| --- | --- |
| Academic Hospital, N (%)^a^ | 45/51 (88%) |
| Profit Type, N (%)^b^ |  |
| Non-profit - private | 29/51 (57%) |
| Non-profit - church | 8/51 (16%) |
| Non-profit – other | 7/51 (14%) |
| Proprietary | 5/51 (10%) |
| Governmental | 2/51 (4%) |
| Bed Size^c^ |  |
| Median (IQR) | 310 (189 - 422) |
| 51-100 beds, N (%) | 5/51 (10%) |
| 101-200 beds, N (%) | 10/51 (20%) |
| >200 beds, N (%) | 36/51 (71%) |
| Pre-Pandemic FTEs Dedicated for Antibiotic Stewardship, Median (IQR)^d^ | 1.2 (0.7-3.5) |
| Abbreviations: IQR, inter-quartile range; full-time equivalents (FTE)  ^a^ Academic hospital status from the American Medical Association’s FREIDA Institution Directory  ^b^ Profit status obtained from data.medicare.gov.  ^c^ Hospital bed size was obtained from the 2015 Michigan Certificate of Need Annual Survey  ^d^ Data are self-reported. An FTE is a unit indicating the workload of a full-time employee. | |
